# White matter hyperintensity distribution differences in aging and neurodegenerative disease cohorts

**DOI:** 10.1016/j.nicl.2022.103204

**Published:** 2022-09-16

**Authors:** Mahsa Dadar, Sawsan Mahmoud, Maryna Zhernovaia, Richard Camicioli, Josefina Maranzano, Simon Duchesne, CCNA Group

**Affiliations:** a Department of Psychiatry, Faculty of Medicine, McGill University, Montreal, QC, Canada; b Department of Anatomy, University of Quebec in Trois-Rivières, Canada; c Department of Medicine, Division of Neurology, University of Alberta, Edmonton, AB, Canada; d Department of Neurology and Neurosurgery, Faculty of Medicine, McGill University, Canada; e Department of Radiology and Nuclear Medicine, Faculty of Medicine, Laval University, Canada

**Keywords:** White matter hyperintensities, Aging, Neurodegenerative disease, Dementia, Magnetic resonance imaging

## Abstract

**Introduction:**

White matter hyperintensities (WMHs) are common magnetic resonance imaging (MRI) findings in the aging population in general, as well as in patients with neurodegenerative diseases. They are known to exacerbate the cognitive deficits and worsen the clinical outcomes in the patients. However, it is not well-understood whether there are disease-specific differences in prevalence and distribution of WMHs in different neurodegenerative disorders.

**Methods:**

Data included 976 participants with cross-sectional T1-weighted and fluid attenuated inversion recovery (FLAIR) MRIs from the Comprehensive Assessment of Neurodegeneration and Dementia (COMPASS-ND) cohort of the Canadian Consortium on Neurodegeneration in Aging (CCNA) with eleven distinct diagnostic groups: cognitively intact elderly (CIE), subjective cognitive impairment (SCI), mild cognitive impairment (MCI), vascular MCI (V-MCI), Alzheimer’s dementia (AD), vascular AD (V-AD), frontotemporal dementia (FTD), Lewy body dementia (LBD), cognitively intact elderly with Parkinson’s disease (PD-CIE), cognitively impaired Parkinson’s disease (PD-CI), and mixed dementias. WMHs were segmented using a previously validated automated technique. WMH volumes in each lobe and hemisphere were compared against matched CIE individuals, as well as each other, and between men and women.

**Results:**

All cognitively impaired diagnostic groups had significantly greater overall WMH volumes than the CIE group. Vascular groups (i.e. V-MCI, V-AD, and mixed dementia) had significantly greater WMH volumes than all other groups, except for FTD, which also had significantly greater WMH volumes than all non-vascular groups. Women tended to have lower WMH burden than men in most groups and regions, controlling for age. The left frontal lobe tended to have a lower WMH burden than the right in all groups. In contrast, the right occipital lobe tended to have greater WMH volumes than the left.

**Conclusions:**

There were distinct differences in WMH prevalence and distribution across diagnostic groups, sexes, and in terms of asymmetry. WMH burden was significantly greater in all neurodegenerative dementia groups, likely encompassing areas exclusively impacted by neurodegeneration as well as areas related to cerebrovascular disease pathology.

## Highlights

• We compared the distribution of WMHs across eleven neurodegenerative disease groups.
• Our results show greater WMH burden in all cognitively impaired and dementia groups compared with controls.
• Women tended to have lower WMH loads than men.
• There were consistent asymmetric patterns in frontal and occipital lobes.

## 1 Introduction

White matter hyperintensities (WMHs) are areas of increased signal on T2-weighted and fluid attenuated inversion recovery (FLAIR) images that commonly occur in elderly individuals as well as patients with neurodegenerative diseases. WMHs have been associated with a multitude of underlying histopathological changes, such as myelin and axonal loss, loss of oligodendrocytes, microglial activation, lipohyalinosis, arteriosclerosis, vessel wall leakage, and collagen deposition in venular walls. Aetiologically, various pathophysiological mechanisms have been proposed, including ischemia, hypoperfusion, increased permeability of the blood–brain barrier, inflammation, degeneration and amyloid angiopathy (Gouw et al., 2010).

Previous studies in the literature have reported differences in WMH volumes between patients with probable Alzheimer’s disease (AD), mild cognitive impairment (MCI), and age matched cognitively healthy controls (Barber et al., 1999, Desmarais et al., 2021, Tosto et al., 2014b). Similar results have been reported in patients with FTD and Lewy body dementia (LBD) (Barber et al., 1999, Desmarais et al., 2021, Varma et al., 2002). The findings have been more inconsistent in patients with PD, with some studies reporting significant differences between PD patients and matched controls, and others not (Dalaker et al., 2009b, Huang et al., 2020, Kandiah et al., 2014, Liu et al., 2021, Sunwoo et al., 2014). In general, later stage PD patients with MCI and dementia tend to have greater levels of WMH, whereas de novo cognitively normal PD patients seem to have similar WMH volumes to controls (Butt et al., 2021, Dadar et al., 2018c, Liu et al., 2021), suggesting a possible link between WMHs and cognitive decline in PD.

Previous studies of neurodegenerative disorders have, in general, focussed on one disorder (e.g. pure AD without comorbid cerebrovascular pathology), limiting their ability to study the overlap between these disorders and recognize vascular contributions across neurodegenerative disorders. Investigating differences in prevalence and distribution of WMHs across different neurodegenerative diseases provides additional insights into the mechanisms through which cerebrovascular and neurodegenerative pathologies interact, possibly shedding light on potential synergistic relationships between theses pathologies.

Previous investigations on sex specific differences in WMHs have reported inconsistent results, some not finding a significant difference (Wen et al., 2009, Ylikoski et al., 1995, Zhuang et al., 2018), some reporting greater WMH burden in women (Alqarni et al., 2021, Sachdev et al., 2009) and others reporting the opposite (Filomena et al., 2015, Geerlings et al., 2010). There have also been a number of studies reporting hemispheric asymmetry of WMH burden, and that this asymmetry might be an important predictor for long-term functional status. (Dhamoon et al., 2017, Low et al., 2019).

Differences in image acquisition protocols and parameters, recruitment criteria, and variability in WMH assessment techniques has hindered the meaningful comparisons of WMH burden across different populations and studies. In essence, previous studies comparing WMH burden across different neurodegenerative diseases with the same image acquisition and WMH assessment methods have generally been performed in relatively small populations and with visual assessments of WMH burden, limiting their statistical power and sensitivity to detect the more subtle differences in WMH burden and distribution across cohorts (Burton et al., 2006, Erkinjuntti et al., 1994, Liu et al., 2021).

Our report addresses these issues. Taking advantage of the Comprehensive Assessment of Neurodegeneration and Dementia (COMPASS-ND) cohort of the Canadian Consortium on Neurodegeneration in Aging (CCNA) and our extensively validated automated WMH segmentation method developed to quantify WMHs in multi-center and multi-scanner studies of aging and neurodegeneration (Dadar et al., 2017), we have compared burden and distribution of WMHs across 11 diagnostic cohorts: cognitively intact elderly (CIE), subjective cognitive impairment (SCI), mild cognitive impairment (MCI), vascular MCI (V-MCI), Alzheimer’s dementia (AD), vascular AD (V-AD), frontotemporal dementia (FTD), Lewy body dementia (LBD), cognitively intact elderly with Parkinson’s disease (PD-CIE), cognitively impaired Parkinson’s disease (PD-CI), and mixed dementias as well as between men and women and left and right hemispheres in each group. To our knowledge, this is the first study that consistently compares the distribution of WMHs across all these neurodegenerative disease populations using a large dataset (N = 976) acquired consistently with a harmonized protocol.

## 2 Methods

### 2.1 Participants

Data included 976 participants from the Comprehensive Assessment of Neurodegeneration and Dementia (COMPASS-ND) cohort of the CCNA, a national initiative to catalyze research on dementia (Chertkow et al., 2019). COMPASS-ND includes cognitively intact elderly subjects as well as patients with various forms of dementia and degenerative disorders and mild memory loss or subjective cognitive concerns. Ethical agreements were obtained at all respective sites. Written informed consent was obtained from all participants.

### 2.2 Clinical diagnoses

Clinical diagnoses were determined by participating clinicians based on longitudinal clinical, screening, and MRI findings and based on standard diagnostic criteria (i.e. diagnosis reappraisal was performed using information from recruitment assessment, screening visit, clinical visit with physician input, and MRI). Diagnostic groups included, AD, CIE, FTD, LBD, MCI, PD-CIE, PD-MCI, PD-Dementia (for this study, PD-MCI and PD-Dementia groups were merged into one PD-CI group), SCI, V-AD, and V-MCI.

**Inclusion/Exclusion Criteria**

Cognitively Intact Elderly (CIE)

1. Age range: 60–90
2. No self-experienced persistent decline in cognitive capacity in comparison with a previously normal status
3. Normal age-, sex-, and education-adjusted performance on standardized cognitive tests

**Subjective Cognitive Impairment (SCI)**

1. Age range: 60–90
2. Self-experienced persistent decline in cognitive capacity in comparison with a previously normal status
3. Normal age-, sex-, and education-adjusted performance on standardized cognitive tests

**Mild Cognitive Impairment due to Alzheimer’s disease (MCI)**

1. Age range: 60–90
2. Must meet National Institute on Aging Alzheimer’s Association (NIA-AA) criteria for amnestic or multi-domain mild cognitive impairment (Albert et al., 2011)
3. Exclusion criteria: abnormality on MRI (e.g. presence of significant cerebrovascular disease, mass lesion) or blood work (vitamin B12 deficiency, hypothyroidism, chronic kidney disease)

**Vascular MCI (V-MCI)**

1. Age range: 60–90
2. Must meet NIA-AA criteria for amnestic or multi-domain mild cognitive impairment (Albert et al., 2011)
3. No history of previous symptomatic stroke (asymptomatic MRI or CT or evidence of silent brain infarction is not an exclusion criterion)
4. CT or MRI showing either: a) 2 or more supratentorial silent brain infarcts, or b) extensive white matter disease defined as score ≥2 on the ARWMC scale

**Mixed Dementia (Mixed)**

1. Age range: 50–90
2. Must meet NIA-AA criteria for mixed etiology dementia (McKhann et al., 2011)
3. Non-AD causes of dementia may be present

**Alzheimer’s Disease (AD)**

1. Age range: 50–90
2. Must meet NIA-AA criteria for Alzheimer’s disease (McKhann et al., 2011)
3. Non-AD causes of dementia ruled out by standard work up (including imaging and blood work)

**Vascular Alzheimer’s Disease (V-AD)**

1. Age range: 50–90
2. Must meet NIA-AA criteria for Alzheimer’s disease as well as mixed etiology dementia (McKhann et al., 2011)

**Lewy Body Dementia (LBD)**

1. Age range: 50–90
2. Subject meets LBD criteria from McKeith et al., 2017 (McKeith et al., 2017)

**Parkinson’s Disease Dementia (PD-Dementia)**

1. Age range: 50–90
2. Subject meets the Movement Disorder Society (MDS) core clinical criteria for Parkinson’s disease dementia (Dubois et al., 2007, Emre et al., 2007, Postuma et al., 2015)

**Mild Cognitive Impairment in Parkinson’s Disease (PD-MCI)**

1. Age range: 50–90
2. Subject meets the MDS core clinical criteria for Mild Cognitive Impairment in Parkinson’s Disease (Litvan et al., 2012)

**Parkinson’s Disease with No Cognitive Impairment (PD-CIE)**

1. Age range: 50–90
2. Subject meets the MDS core clinical criteria for Parkinson’s disease with no cognitive impairment (Postuma et al., 2015)

**Frontotemporal dementia (FTD)**

1. Age range: 50–90
2. Subject meets the FTD subtype criteria for potential or probable Progressive Supranuclear Palsy (PSP) (Litvan et al., 1996), behavioural variant Frontotemporal Dementia (bvFTD)(Rascovsky et al., 2011), Corticobasal Syndrome (CBS) (Armstrong et al., 2013), or Primary Progressive Aphasia (PPA) (Mesulam, 2001)

### 2.3 MRI data

All CCNA participants were scanned using the Canadian Dementia Imaging Protocol, a harmonized MRI protocol designed to reduce inter-scanner variability in multi-centric studies (Duchesne et al., 2019). The following sequences were used to detect WMHs:

- 3D isotropic T1w scans (voxel size = 1.0 × 1.0 × 1.0 mm^3^) with an acceleration factor of 2 (Siemens: MP‐RAGE‐PAT: 2; GE: IR‐FSPGR‐ASSET 1.5; Philips: TFE‐Sense: 2)
- 2D Fluid attenuated inversion recovery (T2w‐FLAIR) images (voxel size = 0.9 × 0.9 × 3 mm^3^), fat saturation, and an acceleration factor of 2.

Table 1 shows the acquisition parameters for each sequence and scanner manufacturer. A detailed description, exam cards, and operators' manual are publicly available at: www.cdip-pcid.ca.

**Table 1:** Acquisition parameters of the CDIP protocol.

Sequence	Scanner Model	Resolution (mm^3^)	TR (msec)	TE (msec)	TI (msec)	Flip Angle
T1w	GE	1.0 × 1.0 × 1.0	6.7	2.9	400	11
	Philips	1.0 × 1.0 × 1.0	7.3	3.3	935	9
	Siemens	1.0 × 1.0 × 1.0	2300	2.98	900	9

FLAIR	GE	0.94 × 0.94 × 3.0	9000	140	2500	125
	Philips	0.94 × 0.94 × 3.0	9000	125	2500	150
	Siemens	0.94 × 0.94 × 3.0	9000	123	2500	165

TR: repetition time; TE: echo time; TI: inversion time.

### 2.4 MRI preprocessing

All images were pre-processed using the following steps: image denoising (Coupe et al., 2008), intensity non-uniformity correction (Sled et al., 1998), and image intensity normalization into range 0–100. FLAIR images were co-registered (rigid registration, 6 parameters) to the T1w images with a mutual information cost function. The T1w scans were also linearly (Dadar et al., 2018a) and nonlinearly (Avants et al., 2008) registered to the MNI-ICBM152-2009c unbiased symmetric average template (Manera et al., 2020). Brain extraction was performed using BEaST, a robust brain extraction method from T1-weighted images built on a multi-resolution patch-based framework (Eskildsen et al., 2012).

### 2.5 WMH segmentations

A previously validated segmentation method was used to segment WMHs (Dadar et al., 2017). The technique employs a set of location and intensity features in combination with a random forests classifier to detect WMHs using T1w and FLAIR images. The WMH segmentation tool was retrained and evaluated using data from the CCNA to ensure the accuracy of the automated WMH segmentations. The training library consisted of manual, expert segmentations of WMHs from 60 participants from the CCNA. These cases were selected from different sites and disease groups to ensure generalizability of the performance of the model. WMHs were automatically segmented for all participants in native FLAIR space.

### 2.6 Quality control

All preprocessed and registered images were visually assessed for quality control (presence of imaging artifacts, failure in registrations). WMH segmentations were also visually assessed for missing hyperintensities or over-segmentation. Either failure resulted in the participant being removed from the analyses (N = 9). All MRI processing, segmentation and quality control steps were blinded to clinical outcomes. Note that the provided data was already quality controlled by the CCNA imaging platform for presence of imaging artifacts, and only scans that had passed this quality control step were collected and used for this study. In final, 976 participants’ MRIs were entered in our analysis (see Table 2 for details).

**Table 2:** Demographic characteristics and WMH volumes (normalized values in cubic centimetres) for the participants used in this study.

Diagnosis	Total	N Female	Male	Age	Total	Frontal	WMHs Parietal	Temporal	Occipital
AD	88	37	51	74.54 ± 7.77	13.81 ± 9.58	6.13 ± 5.10	3.97 ± 3.75	1.69 ± 0.93	2.01 ± 1.15
CIE	105	78	27	69.99 ± 6.14	8.81 ± 6.57	4.14 ± 3.20	2.21 ± 2.35	1.20 ± 0.91	1.24 ± 0.88
FTD	37	20	17	65.65 ± 8.67	17.53 ± 12.57	8.06 ± 6.33	5.56 ± 5.14	1.81 ± 1.33	2.07 ± 1.33
LBD	26	3	23	72.49 ± 8.02	17.59 ± 13.52	7.00 ± 4.72	5.74 ± 5.73	2.11 ± 2.37	2.70 ± 1.93
MCI	264	112	152	72.09 ± 6.55	11.44 ± 10.01	5.34 ± 5.27	3.11 ± 3.96	1.40 ± 0.93	1.57 ± 1.08
Mixed	40	16	24	78.87 ± 6.45	35.28 ± 20.70	18.43 ± 11.13	10.55 ± 8.29	2.95 ± 1.90	3.31 ± 1.80
PD-CIE	76	35	41	67.05 ± 7.37	9.97 ± 9.41	4.90 ± 4.83	2.48 ± 3.56	1.24 ± 0.90	1.33 ± 0.96
PD-CI	51	7	44	72.70 ± 7.68	19.16 ± 18.52	8.99 ± 9.98	5.74 ± 6.86	2.14 ± 1.53	2.26 ± 1.48
SCI	132	98	34	70.55 ± 6.02	10.86 ± 9.21	5.63 ± 4.68	2.89 ± 3.73	1.25 ± 0.95	1.07 ± 3.85
V-AD	24	14	10	77.74 ± 7.17	28.55 ± 17.17	12.49 ± 10.29	9.40 ± 6.17	2.88 ± 1.29	3.72 ± 2.10
V-MCI	133	59	74	76.35 ± 6.27	29.21 ± 17.54	14.31 ± 9.58	9.34 ± 6.94	2.99 ± 1.75	2.53 ± 1.81

### 2.7 WMH segmentations and volumes

The performance of the trained model was also evaluated against the manual expert labels in the training/validation subset (N = 60 participants), through 10-fold cross validation. Results accuracy was high (mean Dice similarity index = 0.80 ± 0.15).

*Volumetric WMH Measures*: All WMH masks were registered to the MNI-ICBM152 average template by concatenating the FLAIR-to-T1w and T1w-to-template transformations. The WMH volumes were calculated after registration of the native masks to the MNI-ICBM152 average template and thus normalized for differences in head size to enable population comparisons. WMH volumes for left and right frontal, parietal, temporal, and occipital lobes as well as the entire brain were calculated based on Hammers atlas (Dadar et al., 2018b, Hammers et al., 2003). WMH volumes were also log-transformed to achieve normal distribution.

**Fig. 1:**
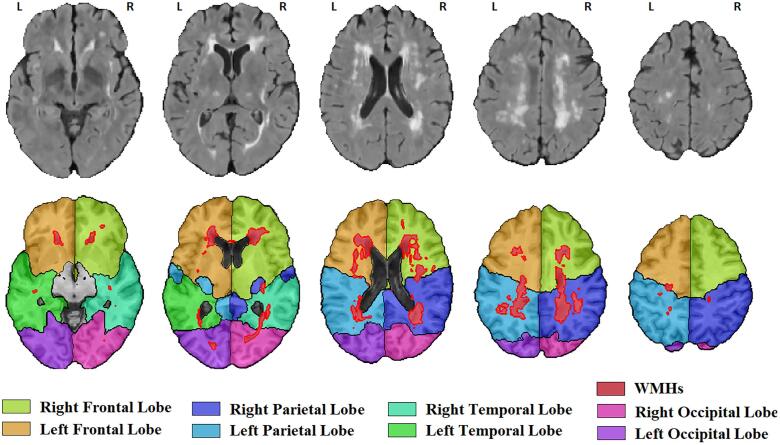
WMH segmentations and separation into left and right frontal, parietal, temporal, and occipital lobes.

Fig. 1 shows an example of the segmented WMHs and their separation into left and right frontal, parietal, temporal, and occipital lobes for one case.

*WMH Distribution Maps*: To obtain comparable voxel-wise WMH distribution maps, we nonlinearly registered all WMH masks to MNI-ICBM152-2009c template by concatenating linear FLAIR-to-T1w, linear T1w-to-ICBM, and nonlinear T1w-to-ICBM registration transformations.

### 2.8 Statistical analysis

The following linear regression models were used to assess differences between WMH volumes across different diagnoses and regions:

WMHVolume∼1+Cohort+Age+Sex

where *WMH Volume* indicates total or lobar WMH burden and *Cohort* is the variable of interest indicating the contrast between each disease cohort and the reference (CIE) group. Similarly, pairwise regression models were run to assess differences between each diagnostic group pair.

The following regression models were used to assess sex differences in WMH burden in each group:

WMHVolume∼1+Age+Sex

In these models, the variable of interest was *Sex*, contrasting women against men (self-reported sex at birth). The models were run separately for each diagnostic group. Paired t-tests were used to assess asymmetric differences between WMH volumes in the left and right hemispheres, contrasting the left hemisphere against the right. WMH volumes were normalized by lobe volumes in these analyses to ensure that slight differences in lobe volumes did not impact results.

WMH volumes were z-scored prior to the regression analyses. All results were corrected for multiple comparisons using false discovery rate (FDR) method with a significant threshold of 0.05. All statistical analyses were performed using MATLAB version 2021a.

## 3 Data and code availability statement

For information on availability and access to CCNA data, see http://ccna.dev.simalam.ca/compass-nd-study/. The WMH segmentation pipeline used is publicly available at http://nist.mni.mcgill.ca/white-matter-hyperintensities/.

## 4 Results

Table 2 summarizes the WMH volumes (normalized values in cubic centimetres) in each diagnostic group.

**Fig. 2:**
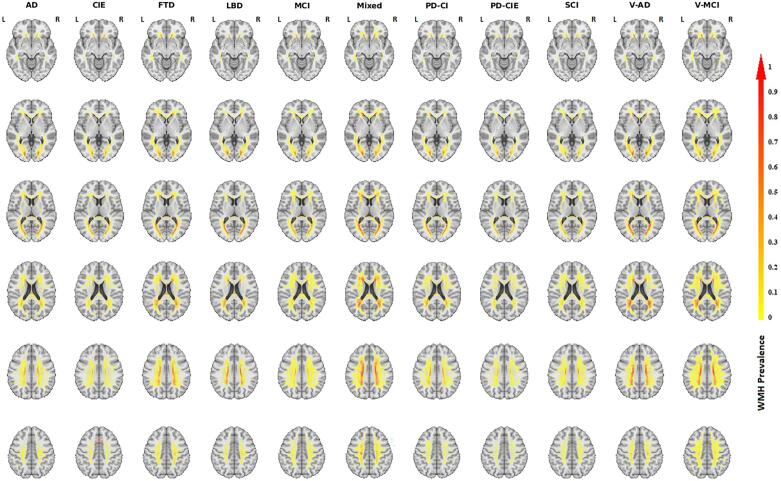
Voxel-wise WMH prevalence maps for each diagnostic group. The color bar indicates the proportion of the subjects in each cohort that had WMHs at each specific voxel location.

Fig. 2 shows the voxel-wise WMH distribution maps for each diagnostic group. WMHs were more prevalent in the periventricular regions for all groups, with the vascular groups (V-MCI, V-AD, and Mixed) having the highest WMH prevalence (see also Table 2). The FTD group also had high WMH prevalence, particularly considering that it included patients that were younger than all other diagnostic groups (mean age = 65.65). Fig. S.1 in the supplementary materials shows the same distribution maps, excluding voxels that had information for<5 subjects in each cohort. The percentage of total white matter (in the MNI atlas) covered by WMHs in at least 5 % of the subjects (or 5 subjects) in each group was 2.93 % (3.02 %) for the CIE group, 5.27 % (4.42 %) for the AD group, 10.06 % (4.03 %) for the FTD group, 6.46 % (2.36 %) for the LBD group, 3.51 % (6.27 %) for the MCI group, 16.12 % (9.30 %) for the Mixed dementia group, 5.69 % (3.42 %) for the PD-CI group, 2.71 % (2.19 %) for the PD-CIE group, 3.10 % (4.16 %) for the SCI group, 10.36 % (4.56 %) for the V-AD group, and 11.85 % (14.33 %) for the V-MCI group.

### 4.1 WMH volumes analyses

**Fig. 3:**
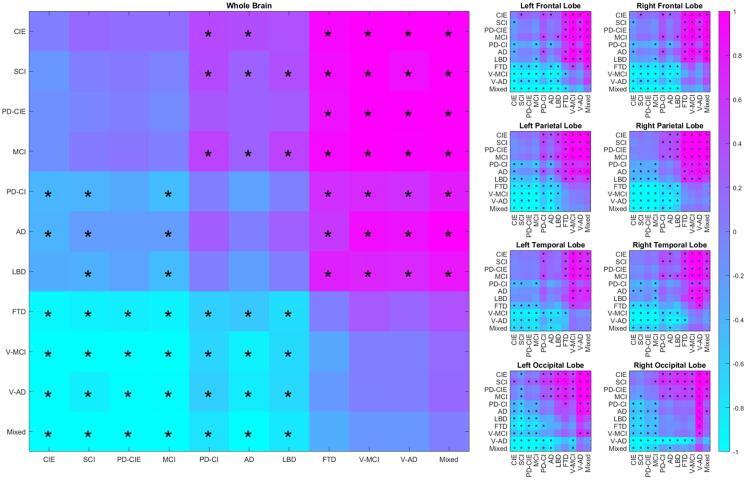
Regional and whole brain group differences in WMH volumes for each diagnostic pair. Colors indicate model β estimates. Significant differences after correction for multiple comparisons are marked with *.

Table 3 shows the differences in total and regional WMH volumes for each group, contrasted against the CIE, and controlling for age and sex. The t statistic values contrast each diagnostic group versus CIE; i.e. a positive value indicates higher WMH in the diagnostic group than the CIE group. Fig. 3 shows model β estimates for pairwise group differences for each diagnostic group pair and region. Significant differences after correction for multiple comparisons are marked with *.

**Table 3:** Total and regional WMH volume differences across diagnostic cohorts (with the CIE group used as reference for the comparisons), controlling for age and sex. Values represent T statistics and uncorrected P values. Significant differences after FDR correction are shown in bold font.

Region	Whole Brain	Left Frontal Lobe	Right Frontal Lobe	Left Parietal Lobe	Right Parietal Lobe	Left Temporal Lobe	Right Temporal Lobe	Left Occipital Lobe	Right Occipital Lobe
AD	**3.32, <0.001**	**2.26, 0.02**	1.76, 0.07	**2.15, 0.03**	**3.14, 0.002**	1.43, 0.15	**2.12, 0.03**	**2.16, 0.03**	**3.22, 0.001**
FTD	**7.15, <0.001**	**5.95, <0.001**	**6.34, <0.001**	**5.99, <0.001**	**6.80, <0.001**	**3.19, <0.001**	**4.44, <0.001**	**4.67, <0.001**	**3.69, <0.001**
LBD	**3.48, <0.001**	**2.52, 0.01**	**2.70, 0.007**	**3.56, <0.001**	**3.34, <0.001**	1.26, 0.20	1.93, 0.05	**3.30, <0.001**	**3.16, 0.002**
MCI	1.53, 0.13	1.38, 0.16	1.14, 0.26	0.51, 0.61	1.07, 0.28	0.19, 0.85	0.25, 0.80	−0.15, 0.88	1.35, 0.18
Mixed	**9.35, <0.001**	**10.27, <0.001**	**9.44, <0.001**	**8.13, <0.001**	**7.55, <0.001**	**5.21, <0.001**	**5.06, <0.001**	**6.33, <0.001**	**4.71, <0.001**
PD-CI	**4.51, <0.001**	**4.41, <0.001**	**4.08, <0.001**	**4.18, <0.001**	**3.30, 0.001**	**2.99, 0.003**	**3.33, 0.001**	1.99, 0.04	**3.36, 0.001**
PD-CIE	1.64, 0.10	**2.42, 0.01**	1.77, 0.08	1.47, 0.14	0.45, 0.65	0.52, 0.60	1.02, 0.30	0.33, 0.74	0.77, 0.44
SCI	2.04, 0.04	**2.88, 0.004**	**2.70, 0.007**	1.48, 0.14	1.29, 0.19	0.51, 0.60	0.04, 0.97	−1.95, 0.05	−0.99, 1.32
V-AD	**6.82, <0.001**	**5.70, <0.001**	**5.23, <0.001**	**6.08, <0.001**	**6.83, <0.001**	**4.14, <0.001**	**5.55, <0.001**	**5.90, <0.001**	**6.60, <0.001**
V-MCI	**12.00, <0.001**	**12.07, <0.001**	**11.67, <0.001**	**10.62, <0.001**	**10.92, <0.001**	**8.43, <0.001**	**8.97, <0.001**	**4.19, <0.001**	**5.05, <0.001**

The CIE group had significantly lower total WMH volumes than all cognitively impaired and dementia groups, except for the MCI group, likely since MCI participants with WMHs were classified in the vascular MCI (V-MCI) category. As expected, V-MCI, V-AD, and Mixed groups had significantly higher WMH volumes than the CIE group in all regions. FTD group also had significantly greater WMH volumes than the CIE group in all regions. The (non-vascular) AD group also had significantly greater WMH volumes than the CIE group overall and in left frontal, bilateral parietal and occipital, and right temporal lobes. The LBD group had significantly greater WMH volumes than the CIE group overall and in bilateral frontal, parietal, and occipital lobes. The cognitively impaired PD (PD-CI) group had significantly greater WMH volumes than the CIE group overall and in all regions, except for left occipital lobe for which the significance did not survive correction for multiple comparisons. Finally, the SCI group had significantly greater WMH volumes than the CIE group in bilateral frontal lobes.

In the pairwise comparisons, vascular groups (i.e. V-MCI, V-AD, and Mixed) had significantly greater WMH volumes than all other groups, except for FTD, which also had significantly greater WMH volumes than all non-vascular groups.

Table 4 shows the differences in total and regional WMH volumes between men and women, controlling for age. The t statistic values contrast women versus men, i.e. a positive value indicates higher WMH in women than men, and vice versa. Overall, women tended to have lower WMH burden than men in most groups and regions, controlling for age. In the CIE group, women had significantly lower WMH volumes for whole brain WMHs, as well as right parietal and temporal lobes. In the FTD group, women had significantly lower WMH volumes for whole brain WMHs as well as right frontal lobe and left parietal and occipital lobes. In the MCI, V-MCI, and PD-CIE groups, women had significantly lower WMH volumes for bilateral occipital lobes.

**Table 4:** Total and regional WMH volume differences between men and women in each diagnostic cohort. Values represent T statistics and uncorrected P values. Significant differences after FDR correction are shown in bold font.

Region	Whole Brain	Left Frontal Lobe	Right Frontal Lobe	Left Parietal Lobe	Right Parietal Lobe	Left Temporal Lobe	Right Temporal Lobe	Left Occipital Lobe	Right Occipital Lobe
AD	0.70, 0.48	1.79, 0.07	1.33, 0.18	0.76, 0.45	0.42, 0.67	0.43, 0.67	−0.49, 0.62	−0.68, 0.50	−1.78, 0.08
CIE	**−2.83, 0.006**	−2.02, 0.04	−1.91, 0.06	−1.90, 0.06	**−3.00, 0.003**	−1.95, 0.05	**−3.17, 0.002**	−1.72, 0.09	−2.00, 0.04
FTD	**−3.16, 0.003**	−2.40, 0.02	**−2.88, 0.007**	**−3.37, 0.002**	−2.10, 0.04	−1.73, 0.09	−1.86, 0.07	**−2.92, 0.006**	−1.37, 0.18
LBD	−1.17, 0.02	−1.04, 0.30	−0.85, 0.40	−0.66, 0.51	−1.08, 0.29	−1.63, 0.11	−1.46, 0.15	−0.88, 0.38	−1.36, 0.18
MCI	−0.03, 0.97	1.68, 0.09	0.78, 0.43	−0.16, 0.87	0.20, 0.83	−1.11, 0.26	0.05, 0.96	**−3.28, 0.001**	**−2.69, 0.008**
Mixed	−0.64, 0.52	0.38, 0.70	−0.64, 0.53	−0.89, 0.38	−1.18, 0.24	−2.34, 0.02	−1.72, 0.09	−2.57, 0.01	−1.48, 0.14
PD-CI	−0.50, 0.62	−0.23, 0.82	0.03, 0.97	−1.09, 0.28	−1.01, 0.31	−0.48, 0.63	−0.46, 0.65	−0.05, 0.96	−0.30, 0.76
PD-CIE	−1.56, 0.12	−0.58, 0.56	−0.32, 0.75	−1.10, 0.27	−1.33, 0.18	−1.80, 0.07	**−2.74, 0.008**	**−2.78, 0.007**	**−3.60, 0.001**
SCI	−0.08, 0.94	0.68, 0.50	0.96, 0.33	0.10, 0.92	0.45, 0.65	−1.75, 0.08	−2.10, 0.03	−2.58, 0.01	−2.56, 0.01
V-AD	−1.02, 0.32	−0.02, 0.98	−0.25, 0.80	−0.40, 0.69	−1.32, 0.20	0.35, 0.73	−0.78, 0.44	−1.95, 0.06	−2.15, 0.04
V-MCI	−2.07, 0.04	−1.13, 0.25	−0.68, 0.49	−2.65, 0.009	−2.18, 0.03	−2.29, 0.02	−2.52, 0.01	**−3.76, <0.001**	**−2.85, 0.005**

**Fig. 4:**
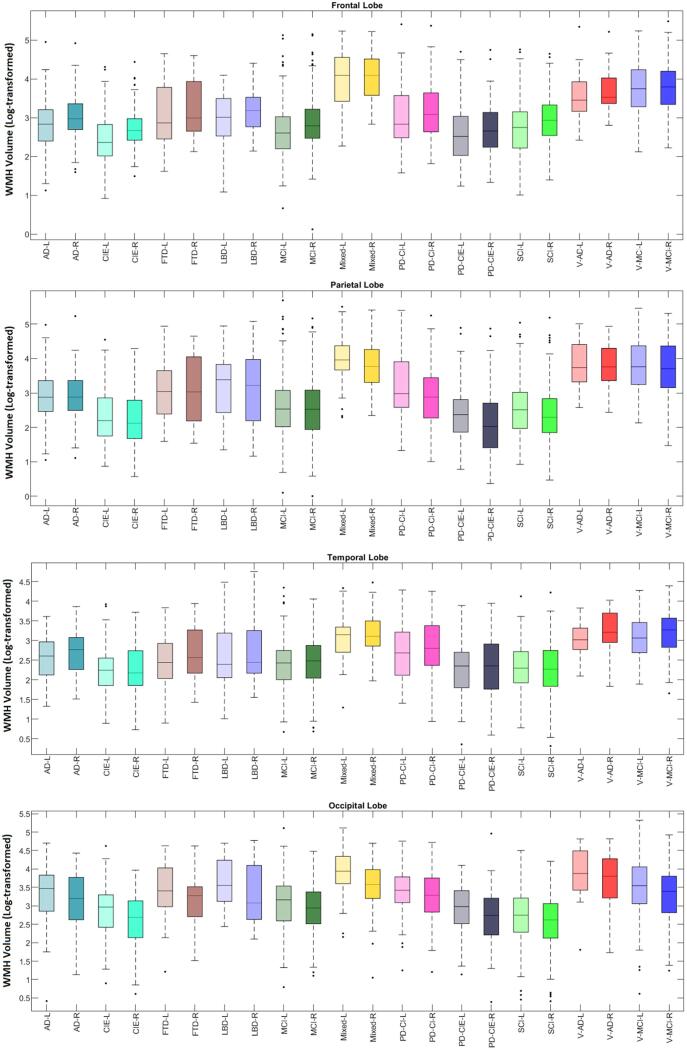
Boxplots of WMH volumes for each diagnostic group, lobe, and hemisphere. L = Left. R = Right.

Table 5 shows the asymmetrical differences between left and right hemispheres (paired t-tests). The t statistic values contrast left versus right, i.e. a positive value indicates higher WMH in the left hemisphere than the right. Fig. 4 shows boxplots of the normalized WMH values for each hemisphere, lobe, and diagnostic group. The left frontal lobe had lower WMH burden than the right in all groups, except for V-AD and mixed cohorts. In contrast, the right occipital lobe tended to have greater WMH volumes than the left in all groups, except for PD-CI and V-AD cohorts. CIE, PD-CI and PD-CIE, and SCI groups had significantly higher WMH volumes in the left parietal lobe, and AD, FTD, LBD, V-MCI, and V-AD groups had significantly greater WMH volumes in the right temporal lobe.

**Table 5:** WMH volume differences between left and right hemispheres in each diagnostic cohort. Values represent T statistics and uncorrected P values. Significant differences after FDR correction are shown in bold font.

Region	Frontal Lobe	Parietal Lobe	Temporal Lobe	Occipital Lobe
AD	**−5.29, <0.001**	−0.58, 0.56	**−3.37, 0.001**	**2.30, 0.02**
CIE	**−11.39, <0.001**	**3.21, 0.002**	−0.51, 0.61	**5.71, <0.001**
FTD	**−4.60, <0.001**	−0.59, 0.56	**−2.75, 0.01**	**3.21, 0.003**
LBD	**−3.45, 0.002**	2.29, 0.03	**−2.75, 0.01**	**3.22, 0.004**
MCI	**−13.45, <0.001**	1.97, 0.05	−2.09, 0.04	**4.82, <0.001**
Mixed	−0.69, 0.49	2.18, 0.04	−1.52, 0.14	**4.84, <0.001**
PD-CI	**−4.74, <0.001**	**4.28, <0.001**	−1.94, 0.06	1.30, 0.20
PD-CIE	**−6.43, <0.001**	**5.63, <0.001**	−1.82, 0.07	**4.14, <0.001**
SCI	**−8.18, <0.001**	**3.70, <0.001**	1.23, 0.22	**4.28, <0.001**
V-AD	−2.28, 0.03	−0.63, 0.54	**−2.69, 0.01**	1.74, 0.10
V-MCI	**−4.85, <0.001**	1.25, 0.21	**−3.63, <0.001**	**3.90, <0.001**

## 5 Discussion

In this study, we compared the distribution of WMHs across eleven distinct neurodegenerative disease diagnostic groups using COMPASS-ND dataset from the CCNA acquired consistently with a harmonized protocol. Our results showed significantly greater WMH burden in all cognitively impaired and dementia groups compared with matched controls, whereas the cognitively intact PD (PD-CI) and SCI groups had slightly higher but non-significant differences.

As expected, the highest burden of WMHs was found in the mixed dementia group, followed by V-MCI and V-AD (Table 1). In patient groups that were not diagnosed with comorbid cerebrovascular pathology, the PD-CI, LBD, and FTD groups had the highest WMH volumes. Interestingly, even the AD group not diagnosed with comorbid vascular pathology had significantly greater WMH burden than the controls, though this difference was much smaller (Table 1, Table 2). The higher burden of WMHs in MCI (Dadar et al., 2018b, DeCarli et al., 2001, Lopez et al., 2003) and AD patients (Burton et al., 2006, Capizzano et al., 2004, Dubois et al., 2014, Huynh et al., 2021, Tosto et al., 2014a) is relatively well established in the literature, whereas the results for other neurodegenerative disease cohorts have been more heterogeneous (Liu et al., 2021).

With regards to links to the underlying proteinopathy, several studies have shown associations between Aβ burden and WMH volume both cross-sectionally and longitudinally in aging, MCI, and AD populations (Graff-Radford et al., 2019, Grimmer et al., 2012, Moscoso et al., 2020, Dadar et al., 2020a, Gurol et al., 2006) as well as in PD (Compta et al., 2016, Dadar et al., 2021), suggesting additive or synergistic associations between the two processes. The blood supply and metabolism disturbances due to hypoperfusion and ischemic changes associated with aging and vascular risk factors could cause neuronal injury and acceleration in over-production and reduction in clearance of Aβ, creating a chain of events that lead to the progressive cognitive deficits and neurodegeneration that characterize AD (De la Torre, 2000). Aβ deposition could also increase WMH burden by accelerating processes that are not necessarily vascular in nature, including neuroinflammation, reactive oxygen species production, and oxidative stress (Scott et al., 2015). In such cases, an initial rise in Aβ would damage the WM, which in turn would elevate Aβ levels, leading to more WM damage in a cyclical process.

We found significantly greater overall and regional WMH volumes in the PD-CI and LBD groups than the CIE, but not in PD-CIE. In addition, while the PD-CI group had higher WMH volumes than the PD-CIE group (mean values of 19.16 cm^3^ versus 9.97 cm^3^ for PD-CI and PD-CIE, respectively, t stat = 1.9, uncorrected p value = 0.05), the difference did not survive correction for multiple comparisons (Fig. 3). Similar to our findings, while studies in later stage PD patients generally report higher levels of WMHs (Liu et al., 2021, Mak et al., 2015, Piccini et al., 1995), studies investigating WMH differences in cognitively normal PD patients have not found significant differences between the patients and age matched controls (Dadar et al., 2018c, Dalaker et al., 2009a). Similarly, while some studies with smaller sample sizes have reported no differences in WMH burden between PD patients with dementia or LBD patients and healthy controls (Burton et al., 2006), others have found such significant differences (Barber et al., 1999), suggesting that larger cohorts are necessary to distinguish the more subtle differences (Butt et al., 2021).

Similar to other studies in the literature, FTD patients had significantly greater WMH burden than the CIE group (Desmarais et al., 2021, Huynh et al., 2021, Varma et al., 2002). In fact, the FTD group was the only non-vascular diagnostic group that had significantly greater overall WMH volumes than all other groups, controlling for age and sex (Fig. 3). This is particularly interesting, given that the FTD group was also the youngest group (Table 1), and older age is known to be the most significant correlate of WMHs. This might be in part due to the fact that high WMH burden can be observed in FTD patients in absence of significant vascular risk factors and pathology, possibly due to other pathological processes related to genetic mutations (Caroppo et al., 2014, Desmarais et al., 2021, Sudre et al., 2019, Woollacott et al., 2018). The FTD group included mostly PPA (N = 19) and bvFTD (N = 12) patients, and controlling for age and sex, WMH burden was not different across the two groups. Unfortunately, our sample did not have genetic status information available, limiting our ability to investigate whether the FTD patients were carriers of specific mutations (e.g. progranulin gene), previously linked to increased WMH burden in genetic forms of FTD (Caroppo et al., 2014, Desmarais et al., 2021, Sudre et al., 2019, Woollacott et al., 2018).

The SCI group had significantly greater WMH volumes in bilateral frontal lobes than the CIE (p < 0.007). While marginally higher (mean values of 10.86 cm^3^ versus 8.81 cm^3^ for SCI and CIE groups, respectively, *t*-stat = 2.04, uncorrected p value = 0.04), the overall WMH burden difference was not significant after correction for multiple comparisons. Previous reports on the association between WMHs and SCI have been varied. Van Rooden et al. reported significantly greater WMH volumes and Fazekas scores in SCI subjects compared to controls (van Rooden et al., 2018), while Caillund et al. did not find such differences (Caillaud et al., 2020). They did however report significant associations between WMHs and executive function in SCI (Caillaud et al., 2020). Similarly, Benedictus et al. linked presence of WMHs to future cognitive decline and progression to MCI or dementia (Benedictus et al., 2015).

While there are sex specific differences in causes and consequences of WMHs (Kumar and McCullough, 2021), the reports on sex differences in prevalence of WMHs have been inconsistent, with some studies not finding a significant difference (Wen et al., 2009, Ylikoski et al., 1995, Zhuang et al., 2018), some reporting greater WMH burden in women (Alqarni et al., 2021, Sachdev et al., 2009) and others reporting the opposite (Filomena et al., 2015, Geerlings et al., 2010). According to Simon et al., the greater WMH burden reported in women might be confounded by age, and be biased by premature death in men (which might be associated with cerebrovascular disease) (Simoni et al., 2012). Another confounding factor might be birth cohort differences, since more recently born cohorts are known to have healthier brain structures and lower WMH volumes (Turcotte et al., 2022), and these differences might have a sex-related predilection. In the present cohort, women tended to have lower WMH volumes than men in general, controlling for age. In the CIE group, women had significantly lower WMH volumes for whole brain WMHs, as well as right parietal and temporal lobes. In the FTD group, women had significantly lower WMH volumes for whole brain WMHs as well as right frontal lobe and left parietal and occipital lobes. In the MCI, V-MCI, and PD-CIE groups, women had significantly lower WMH volumes for bilateral occipital lobes. Sex specific differences in prevalence of WMHs might also be in part due to differences in prevalence of and susceptibility to underlying associated risk factors such as hypertension, diabetes and obesity, hypercholesterolemia, alcohol abuse, and smoking (Bonberg et al., 2022, Lohner et al., 2022). While we did not have this data available, investigating the prevalence of vascular risk factors in 51,338 Canadians, the Canadian Longitudinal Study of Aging (CLSA) have reported higher prevalence of hypertension, heart disease, diabetes, and midlife obesity in men, whereas women had higher rates of late life obesity (Griffith et al., 2019). Other studies have also reported higher levels of smoking (current and former) in Canadian men (Tanuseputro et al., 2003). These differences might in turn lead to differences in prevalence of WMHs between men and women. However, in terms of the underlying risk factors, socioeconomic status, and more importantly, underlying neurodegenerative pathologies, CLSA might not be representative of the CCNA population, and further investigations into associated vascular risk factors and co-morbidities are necessary to disentangle sex specific differences in prevalence of WMHs in the CCNA.

Regarding asymmetry, we found an overall greater burden of WMHs in right frontal and left occipital lobes (Fig. 4). CIE, PD-CI and PD-CIE, and SCI groups had significantly higher WMH volumes in the left parietal lobe, and AD, FTD, LBD, V-MCI, and V-AD groups had significantly greater WMH volumes in the right temporal lobe. The presence of hemispheric dominance and predominance of WMH burden in right frontal lobe has been previously reported by Dhamoon et al. (2017). They also indicate that regional WMH volume asymmetry might be associated with lower function and functional decline (Dhamoon et al., 2017). Low et al. have also reported left-dominant occipital WMH burden in AD patients which was higher than that observed in MCI and control groups, as well as an association with poorer global cognition, memory, language, and executive functions among cognitively impaired participants (MCI and AD) (Low et al., 2019). There are established regional brain asymmetries in the frontal and occipital lobes which are also phylogenetically evident and have been detected in older primates (Toga and Thompson, 2003). These differences might also indicate differences in the evolutionary development of these lobes, leading to differences in susceptibility to neurodegeneration and presence of white matter pathology. There is evidence for asymmetric pathologic burden in neurodegenerative diseases such as PD (asymmetric distribution of α-synuclein aggregates) and AD (asymmetry in reduction of glucose metabolism, amyloid β pathology, and cortical thinning) (Lubben et al., 2021). Lubben et al. suggest that differences in epigenetic regulation and gene expression between hemispheres might drive asymmetries in cellular distribution, connectivity, neurotransmitters, and protein expression across the brain. These differences may in turn lead to hemispheric susceptibility for disease pathogenesis (Lubben et al., 2021). Further research on asymmetry of the white matter pathology in different neurodegenerative disorders is necessary to elucidate the underlying mechanisms involved in asymmetric WMH patterns.

With regards to limitations, the present study reports on the prevalence and differences in WMHs across different diagnostic groups, sexes, and brain hemispheres in isolation. Future studies are warranted to link these findings to prevalence and distribution of other markers of cerebrovascular disease (such as microbleeds, lacunes, and dilated perivascular spaces), as well as presence and patterns of neurodegeneration and gray matter atrophy. Furthermore, we did not have data on the cardiovascular risk factors such as hypertension, hypercholesterolemia, diabetes, and obesity for this population, and were thus not able to assess the relationships between these risk factors and WMH burden in different diagnostic groups. Future investigations are necessary to assess potential differences in relationships between WMH volume and risk factors in different neurodegenerative disease populations. Due to the large number of analyses performed and sample size considerations, we limited the number of regions assessed to lobes and hemispheres. When sufficiently large sample sizes are available, breaking down the regions further based on distance from the ventricles as well as the basal ganglia such as the work by Sudre et al. 2018 might provide further information about the differences in WMH distribution patterns across different diagnostic groups (Sudre et al., 2018). It also is worthwhile noting that the group definitions in the CCNA might differ from other studies in the literature. For example, CSF or PET biomarker status (e.g. amyloid beta levels for AD and dopamine for LBD) were not used for the diagnoses. Additionally, studies such as the ADNI inclusion/exclusion criteria had a Hachinksi score of less than or equal to 4 as an inclusion criterion (https://adni.loni.usc.edu/wp-content/uploads/2010/09/ADNI_GeneralProceduresManual.pdf), essentially excluding cases with high cerebrovascular pathology burden, whereas the CCNA did not impose such criteria for diagnosis, to allow for inclusion of a group of subjects that are more representative of the normal population.

The image processing and WMH segmentation pipelines used in the present study have been developed and validated for use in multi-center and multi-scanner studies of aging and neurodegenerative disease populations and have been previously used in such applications (Anor et al., 2021, Dadar et al., 2020b, Misquitta et al., 2020, Sanford et al., 2019). In addition to initial validation of the performance of the pipeline against gold standard manual segmentations which showed excellent agreement, manual quality control was performed to ensure the quality of the raw images, registrations, and segmentations. Images were acquired using a harmonized protocol which should negate the need to include scanning site as a variable in our models. To verify this assumption, we repeated all analyses using mixed effect modelling, including site as a categorical random effect, and obtained similar results in terms of effect size and significance.

Differences in image acquisition protocols, recruitment criteria, and WMH assessment techniques hinder reliable comparisons of WMH burden across different populations and studies. Using the COMPASS-ND cohort, acquired consistently with a harmonized protocol, we have compared burden and distribution of WMHs across 11 diagnostic cohorts, showing significantly greater WMH burden in all cognitively impaired and dementia groups compared with the matched controls, as well as asymmetric and sex specific trends. This emphasizes the need for further longitudinal investigations into the impact of WMHs in these neurodegenerative diseases as well as treatment and prevention strategies for vascular risk factors (anti-hypertensive medications, blood sugar management, lipid-lowering treatments, exercise, and lifestyle changes), which might slow down WMH progression and cognitive decline in neurodegenerative disease populations.

In these cohorts (even in cohorts not formally considered as mixed with vascular pathology), WMHs likely encompass areas exclusively impacted by neurodegeneration as well as areas related to non-specific MRI based small-vessel disease pathology. The cohorts formally classified as mixed, V-AD, and V-MCI, represent populations with more striking vascular pathogenic factors; hence the WMH volume of those groups are the highest among all cohorts. Future studies, refining and ranking the vascular pathogenic factors along with the MRI findings (e.g. presence of lacunar infarcts or macroscopic infarcts with clear vascular distribution patterns following vascular arterial territories) could help determine the extent of WMHs exclusively related to neurodegeneration versus small vessel disease and establish which component has a greater impact on cognitive decline and other clinical outcomes.

## Authors contributions

Mahsa Dadar: Study concept and design, analysis of the data, drafting and revision of the manuscript.

Sawsan Mahmoud: Manual Segmentation of WMHs.

Maryna Zhernovaia: Manual Segmentation of WMHs.

Richard Camicioli: Study concept and design, interpretation of the data, revising the manuscript.

Josefina Maranzano: Study concept and design, interpretation of the data, revising the manuscript.

Simon Duchesne: Study concept and design, interpretation of the data, revising the manuscript.

## CRediT authorship contribution statement

**Mahsa Dadar:** Conceptualization, Formal analysis, Methodology, Visualization, Writing – original draft, Writing – review & editing. **Sawsan Mahmoud:** Writing – review & editing. **Maryna Zhernovaia:** Writing – review & editing. **Richard Camicioli:** Conceptualization, Writing – review & editing. **Josefina Maranzano:** Conceptualization, Writing – review & editing. **Simon Duchesne:** Conceptualization, Writing – review & editing.

## Declaration of Competing Interest

The authors declare that they have no known competing financial interests or personal relationships that could have appeared to influence the work reported in this paper.

## Data Availability

WMH data has been shared with the CCNA, and will be provided to all users that obtain CCNA approval.
