# Supplementary material for: White matter hyperintensity distribution differences in aging and neurodegenerative disease cohorts
Source: Neuroimage Clin. 2022 Sep 16;36:103204. doi: 10.1016/j.nicl.2022.103204 (PMC9668605; doi:10.1016/j.nicl.2022.103204)
Supplement: Supplementary Data 1 [file mmc1.docx]

Table 1. Distribution of patients across sites

|  | AD | CIE | FTD | LBD | MCI | Mixed | PD-CI | PD-CIE | SCI | V-AD | V-MCI |
| --- | --- | --- | --- | --- | --- | --- | --- | --- | --- | --- | --- |
| BCT | 0 | 1 | 0 | 0 | 17 | 0 | 0 | 0 | 22 | 0 | 3 |
| BCU | 1 | 0 | 0 | 0 | 8 | 0 | 0 | 0 | 0 | 0 | 0 |
| BRI | 1 | 16 | 0 | 0 | 6 | 2 | 1 | 0 | 1 | 1 | 3 |
| CBH | 8 | 1 | 8 | 4 | 14 | 6 | 1 | 2 | 3 | 2 | 6 |
| CMA | 0 | 0 | 0 | 0 | 0 | 0 | 0 | 0 | 4 | 0 | 0 |
| CSA | 2 | 0 | 0 | 0 | 0 | 0 | 0 | 0 | 1 | 0 | 0 |
| DMC | 1 | 0 | 0 | 0 | 5 | 0 | 0 | 0 | 4 | 1 | 2 |
| HEJ | 5 | 8 | 4 | 2 | 9 | 1 | 0 | 0 | 3 | 0 | 1 |
| HHS | 2 | 0 | 4 | 1 | 0 | 1 | 0 | 1 | 0 | 0 | 1 |
| HMC | 13 | 1 | 6 | 2 | 14 | 0 | 2 | 9 | 0 | 0 | 4 |
| HNB | 1 | 5 | 0 | 0 | 3 | 1 | 0 | 0 | 0 | 1 | 3 |
| IUS | 1 | 1 | 0 | 0 | 3 | 1 | 0 | 0 | 2 | 0 | 2 |
| JGH | 13 | 9 | 0 | 0 | 22 | 10 | 2 | 0 | 4 | 5 | 16 |
| KRC | 1 | 1 | 0 | 0 | 3 | 3 | 0 | 0 | 0 | 0 | 0 |
| LHS | 0 | 0 | 0 | 1 | 0 | 0 | 3 | 1 | 0 | 0 | 0 |
| MND | 0 | 0 | 0 | 0 | 0 | 0 | 1 | 0 | 0 | 0 | 0 |
| MNI | 0 | 0 | 2 | 0 | 0 | 0 | 0 | 0 | 0 | 0 | 0 |
| MUC | 0 | 0 | 0 | 0 | 0 | 0 | 7 | 17 | 0 | 0 | 0 |
| PKD | 13 | 12 | 0 | 0 | 53 | 10 | 0 | 0 | 18 | 7 | 40 |
| PKF | 7 | 3 | 3 | 5 | 2 | 0 | 0 | 0 | 2 | 1 | 0 |
| PKM | 0 | 18 | 0 | 0 | 26 | 0 | 0 | 0 | 23 | 0 | 27 |
| RUH | 3 | 0 | 0 | 0 | 1 | 0 | 4 | 7 | 1 | 1 | 0 |
| SBK | 0 | 5 | 5 | 3 | 4 | 0 | 7 | 5 | 1 | 0 | 1 |
| SPH | 1 | 0 | 0 | 0 | 0 | 1 | 0 | 0 | 0 | 0 | 1 |
| UGM | 0 | 0 | 0 | 0 | 20 | 0 | 0 | 0 | 35 | 0 | 10 |
| UHN | 4 | 2 | 1 | 0 | 1 | 0 | 4 | 8 | 1 | 2 | 0 |
| UOA | 6 | 9 | 3 | 7 | 7 | 1 | 18 | 26 | 0 | 1 | 2 |
| UOW | 0 | 3 | 0 | 0 | 8 | 2 | 0 | 0 | 2 | 0 | 7 |
| VGH | 0 | 0 | 0 | 0 | 19 | 0 | 0 | 0 | 0 | 0 | 3 |
| VIA | 4 | 2 | 1 | 1 | 6 | 1 | 1 | 0 | 2 | 2 | 0 |
| WLU | 1 | 8 | 0 | 0 | 13 | 0 | 0 | 0 | 3 | 0 | 1 |

**
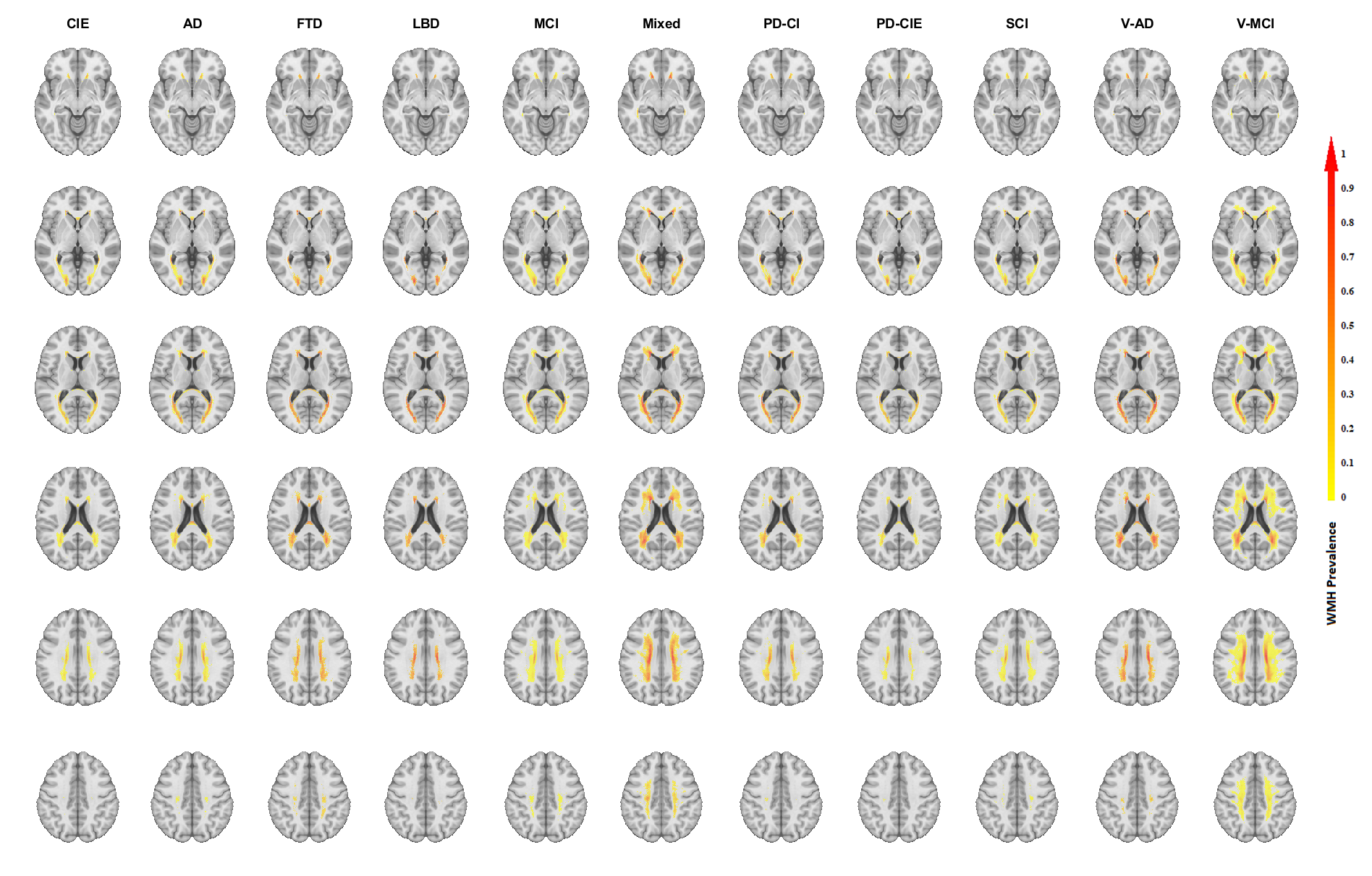
**

Figure S.1. Voxel-wise WMH prevalence maps for each diagnostic group. The color bar indicates the proportion of the subjects in each cohort that had WMHs at each specific voxel location.
